# The current insecticide resistance status of *Anopheles gambiae* (*s.l.*) (Culicidae) in rural and urban areas of Bouaké, Côte d’Ivoire

**DOI:** 10.1186/s13071-018-2702-2

**Published:** 2018-03-02

**Authors:** Dounin Danielle Zoh, Ludovic Phamien Ahoua Alou, Mahama Toure, Cédric Pennetier, Soromane Camara, Dipomin François Traore, Alphonsine Amanan Koffi, Akré Maurice Adja, Ahoua Yapi, Fabrice Chandre

**Affiliations:** 1 Institut Pierre Richet, 01 BP 1500, Bouaké, 01 Côte d’Ivoire; 2 Université Félix Houphouët Boigny, 08 BP 3800, Abidjan, 08 Côte d’Ivoire; 3 Centre d’Entomologie Médicale et Vétérinaire, 27 BP 529, Abidjan, 27 Côte d’Ivoire; 4 MIVEGEC, Institut de Recherche pour le Développement – CNRS - University Montpellier, Délégation Occitanie, 911 Av. Agropolis, Montpellier, France

**Keywords:** Malaria, Insecticide resistance, Metabolic resistance, *Kdr*, *Ace-1*, *An. gambiae* (*s.s.*), *An. coluzzii*

## Abstract

**Background:**

Several studies were carried out in experimental hut station in areas surrounding the city of Bouaké, after the crisis in Côte d’Ivoire. They reported increasing resistance levels to insecticide for malaria transmiting mosquitoes. The present work aims to evaluate the current resistance level of *An. gambiae* **(***s.l*.) in rural and urban areas in the city of Bouaké.

**Methods:**

Larvae of *Anopheles gambiae* (*s.l.*) were collected from five different study sites and reared to adult stages. The resistance status was assessed using the WHO bioassay test kits for adult mosquitoes, with eight insecticides belonging to pyrethroids, organochlorines, carbamates and organophosphates classes. Molecular assays were performed to identify the molecular forms of *An. gambiae* (*s.l.*), the L1014F *kdr* and the *ace-1R* alleles in individual mosquitoes. The synergist PBO was used to investigate the role of enzymes in resistance. Biochemical assays were performed to detect potential increased activities in mixed function oxidase (MFO) levels, non-specific esterases (NSE) and glutathione S-transferases (GST).

**Results:**

High resistance levels to pyrethroids, organochlorines, and carbamates were observed in *Anopheles gambiae* (*s.l.*) from Bouaké. Mortalities ranged between 0 and 73% for the eight tested insecticides. The pre-exposure to PBO restored full or partial susceptibility to pyrethroids in the different sites. The same trend was observed with the carbamates in five sites, but to a lesser extent. With DDT, pre-exposure to PBO did not increase the mortality rate of *An. gambiae* (*s.l.*) from the same sites. Tolerance to organophosphates was observed. An increased activity of NSE and higher level of MFO were found compared to the Kisumu susceptible reference strain. Two molecular forms, S form [(*An. gambiae* (*s.s*)] and M form (*An. coluzzi*) were identified. The *kdr* allele frequencies vary from 85.9 to 99.8% for *An. gambiae* (*s.s.*) and from 81.7 to 99.6% for *An. coluzzii*. The *ace-1R* frequencies vary between 25.6 and 38.8% for *An. gambiae* (*s.s*.) and from 28.6 to 36.7% for *An. coluzzii.*

**Conclusion:**

Resistance to insecticides is widespread within both *An. gambiae* (*s.s.*) and *An. coluzzii*. Two mechanisms of resistance, i.e. metabolic and target-site mutation seemed to largely explain the high resistance level of mosquitoes in Bouaké. Pyrethroid resistance was found exclusively due to the metabolic mechanism.

## Background

The dominant mosquito species responsible for the transmission of malaria parasites in tropical and subtropical Africa include *Anopheles gambiae* (*s.s.*) (S molecular form), *An. coluzzii* (M molecular form), *An. arabiensis* and *An. funestus* [1, 2]. Four classes of insecticides (pyrethroids, organophosphates, carbamates and organochlorines) are the cornerstone of vector control programs [3], but pyrethroids are the only class of insecticide currently recommended by the WHOPES for the treatment of nets because of their irritant and fast-acting properties and their safety for humans [4]. However, resistance to these four classes of chemical insecticides is widespread among *An. gambiae* (*s.l.*) in sub-Saharan Africa [5, 6] and represents a major threat to the effectiveness of malaria vector control strategies based on long-lasting insecticidal nets (LLINs) and indoor residual spraying (IRS) which were the major contributors to the drastic reduction of *Plasmodium falciparum* infection prevalence over the past decade [7]. Thus, the arsenal for managing resistance and providing suitable and sustainable vector control with existing chemicals is becoming seriously limited. To solve the problem, the most advanced strategy could be the use of the combination of two chemicals, presenting different modes of action, into one LLIN [8–11].

In Côte d’Ivoire, the most recent studies carried out in localities surrounding the city of Bouaké showed sympatric populations of *An. gambiae* (*s.s.*) and *An. coluzzii* [12, 13]. These anopheline populations exhibited high resistance to organochlorines, pyrethroids and carbamates based on multifactorial mechanisms (metabolic and target site mutations L1014F *kdr* and *ace-1R* G119S). Insecticide resistance in malaria vectors is a dynamic process in which the resistance level might change quickly and strongly by the mean of selection pressure from both public health and agricultural practices [14–16].

The Bouaké area is characterized by the presence of shallows within and around the city. The use of these shallows for rice farming and vegetable cultivation is accompanied by an outburst of mosquitoes. Indeed, these shallows constitute suitable breeding sites for mosquitoes such as *Anopheles* species [17]. Both crop pest management and public health use insecticides belonging to the same families (e.g. pyrethroids or organophosphates). In addition, since 2010 the National Malaria Control Program (NMCP) has implemented a nationwide distribution of LLINs to scale up the universal coverage of people. The success of malaria control and vector resistance management strategies requires routine insecticide resistance monitoring for malaria vectors, as advised by the WHO Global Plan for Insecticide Resistance Management [18]. This study aims to evaluate and characterize the current resistance of malaria vectors in Bouaké rural and urban areas.

## Methods

### Study area

This study was carried out in three urban districts (Dar Es Salam, Kennedy and N’gattakro) and two villages (Petessou and Allokokro) from Bouaké city in central Côte d’Ivoire.

**Fig. 1:**
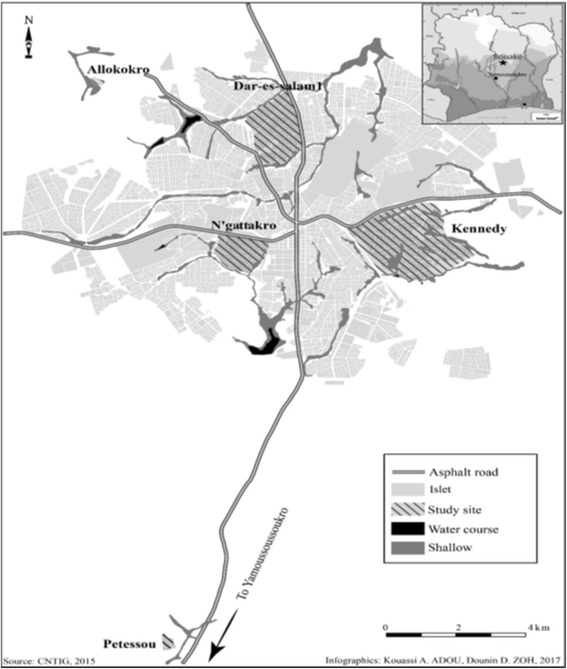
Study sites

The urban districts were crossed by numerous small streams regularly spaced from 300 to 800 m. Thus, the different districts are separated from one another by humid shallows ribbons. As for the villages, Allokokro is bordered by a small marshy area crossed by a permanent river. Regarding Petessou, a permanent watercourse maintains a very large area of shallows and swamps around the village. The shallows present in all the study sites used for rice farming and market gardening, form numerous suitable breeding sites for mosquitoes (Fig. 1).

### Mosquito collection

During the rainy season in August 2014, larvae of *An. gambiae* (*s.l.*) were collected in paddy fields and puddles simultaneously in all study sites i.e. in Allokokro, Petessou, Dar Es Salam, Kennedy and N’gattakro. Larvae were reared at the IPR (Institut Pierre Richet, Bouaké) insectary until adult stages. Test mosquitoes were identified morphologically and only females of *An. gambiae* (*s.l*.) were selected for bioassays. The “Kisumu” strain of *A. gambiae* (*s.l.*) was used as susceptible reference. The “Kisumu” reference strain, susceptible to all insecticides used in this study originated from Kenya.

### WHO susceptibility assays

Bioassays were carried out using the WHO test kits for adult mosquitoes with impregnated papers obtained from the WHO supplier at University Saints Malaysia [19]. Impregnated papers were stored at 4 °C and used no more than three times. Eight insecticides were tested at diagnostic doses as defined by the WHO [19]: three pyrethroids (permethrin 0.75%, deltamethrin 0.05% and α-cypermethrin 0.05%); one organochlorine (DDT 4%); two organophosphates (pirimiphos-methyl 1% and chlorpyrifos-methyl 0.4%) and two carbamates (bendiocarb 0.1% and carbosulfan 0.4%). Tests were performed with batches of 25 unfed females of *An. gambiae* (*s.l*.), 2–4 days-old and using four replicates per concentration. Mosquitoes were exposed to insecticide-treated papers for 1 h. After the exposure period, all mosquitoes were transferred to an observation tube of the test kit, supplied with 10% honey solution and held.

Batches exposed to untreated papers were used as control. Field samples were compared to the susceptible reference strain of *An. gambiae* (*s.s.*) (Kisumu). All control survival specimens (including the susceptible reference mosquito) unexposed to insecticides were stored at -80 °C for biochemical analysis. The samples of mosquitoes exposed to different insecticides were kept individually at -20 °C according to their phenotypic state (dead or alive) for further molecular analysis.

### Bioassays using synergist

In order to assess the involvement of detoxifying enzymes in the resistance to pyrethroids and carbamates, complementary tests with a synergist were performed with a pre-exposition to PBO 4%, an inhibitor of oxidases and to a lesser extent esterases [20]. Unfed females of *An. gambiae* (*s.l.*) aged 2–4 days were pre-exposed to PBO for 1 h before they were exposed to papers impregnated with insecticide. Control mosquitoes were pre-exposed with non-impregnated papers for 1 h. Mosquitoes were transferred to observation tubes, supplied with 10% honey solution and held for 24 h before recording mortality. The same tests were conducted in *An. gambiae* (*s.s.*) susceptible strain (Kisumu). Four replicates of 25 mosquitoes were exposed to each treatment.

### Biochemical assays

Biochemical experiments were carried out by using the procedures outlined by the WHO [21]. As the enzymes degrade rapidly at room temperature, it was essential to always work on ice. Adult mosquitoes from each population were individually homogenized in 200 μl of ice cold distilled water. The homogenate was centrifuged at 14,000× *rpm* for 2 min. Two microplate wells were then each filled with 20 μl of the centrifuged homogenate for the oxidases. The other 4 microplates were filled with 10 μl in 2 replicas for the amount of proteins, and the evaluation of the GST and esterase activities. The different optical densities (OD) were read with defined wavelengths as a function of the enzymatic system to be assayed.

#### Total proteins

Total protein was measured for each mosquito using the method of Bradford [22]. The amount of proteins was determined to serve as a basis for the quantification of enzyme activities. Ten microliters of each homogenate was mixed with 200 μl of a solution of “Coomassie Plus Protein Assay Reagent” half diluted in distilled water (1 volume of protein reagent per 1 volume of water). This solution was held for 5 min at room temperature. The optical density (OD) was read at the end-point at 590 nm.

#### Mixed function oxidase assay

P450 activity was measured following Brogdon et al. [23]. Twenty microliters of homogenate was mixed with 80 μl of potassium phosphate buffer pH 7.2 + 200 μl of tetramethyl benzidine (TMBZ) working solution [(0.012 g TMBZ dissolved in 6 ml methanol and then in 18 ml of sodium acetate buffer pH 5.0) + 25 μl of 3% (*v*/v) H_2_O_2_ solution] in a microplate well. After 30 min incubation at room temperature with a lid, the plate was read at 630 nm as an end-point assay. This assay does not measure the monooxygenase activity, but titrates the amount of bound haem in the mosquito homogenate. Since haem is present in the active site of monooxygenases, major changes in the amount of monooxygenases produce a measurable increase in haem. By using a standard curve of cytochrome C (which also contains bound haem) a crude estimate of the amount of the monooxygenases present was obtained and expressed as equivalent units of cytochrome P450.

#### Glutathione S-transferase assay

Glutathione S-transferase assay activity was performed according to the WHO [21]. Ten microliters of each homogenate was mixed with 200 μl of reduced glutathione (GSH)/1-chloro-2,4 dinitrobenzene (CDNB) working solution [0.060 g of reduced glutathione (GSH) in 20 ml of sodium phosphate buffer 0.1 M pH 6.5 + 0.013 g of CDNB diluted in 1 ml of methanol] in a microplate well. The reaction was read at 340 nm immediately as a kinetic assay for 5 min. An extinction coefficient of 5.76 mM^-1^ (corrected for a path length of 0.6 cm) was used to convert absorbance values to moles of product. GST specific activity was reported as CDNB conjugated μmol product min^-1^ mg^-1^ protein.

#### Esterase assays

Non-specific esterase activity was assessed with two substrates, α- and β-naphthol acetate using the WHO protocol [21]. Ten microliters of each homogenate was mixed with 90 μl of 1% saline phosphate buffer (PBS) (pH 6.5). After 10 min incubation at room temperature, 100 μl of solution [α or β-naphthyl acetate (600 μl of 0.06 M α-naphthyl acetate + 3 ml of 1% PBS buffer (pH 6.5) + 8.4 ml of distilled water)] was added. After incubation for 30 min at room temperature, 100 μl of solution (0.012 g of Fast Garnett Salt + 12 ml distilled water) was added. This solution was again incubated for 10 min at room temperature. The reaction was read immediately at two minutes at 550 nm as the end-point. Esterase specific activity per individual was reported as μmol product.min^-1^.mg protein^-1^.

### Molecular analysis

#### DNA extraction

Genomic DNA was extracted from individual mosquitoes using cetyl trimethyl ammonium bromide (CTAB) 2% as described by Yahouedo et al. [24] and used for PCR analysis to identify sub-species of *An. gambiae* complex, and detect L1014F *kdr* and *ace-1R* G119S mutations.

#### Molecular form detection

Species identification of *An. gambiae* (*s.l.*) was performed by PCR according to Favia et al. [25]. The PCR conditions were 10 min at 94 °C as the initial step, followed by 45 cycles (94 °C for 30 s, 63 °C for 30 s and 72 °C for 30 s). After the last cycle, the products were finally extended for 7 min at 72 °C. Primers used in the PCR were: R5 (5′-GCC AAT CCG AGC TGA TAG CGC-3′), R3 (5′-CGA ATT CTA GGG AGC TCC AG-3′), Mop int (5′-GCC CCT TCC TCG ATG GCA T-3′), B/S int (5′-ACC AAG ATG GTT CGT TGC-3′). Amplified fragments were analyzed on a 1.5% agarose gel.

#### L1014F *kdr* mutation detection

The L1014F *kdr* mutation detection was determined according to Martinez-Torres et al. [26] based on PCR-AS. Two primers, Agd1 (5′-ATA GAT TCC CCG ACC ATG-3′) and Agd2 (5′-AGA CAA GGA TGA TGA ACC-3′) were used to amplify a common sequence of 293 base pairs (bp) in all *An. gambiae* (*s.l.*) mosquitoes. Both specific primers Agd3 (5′-AAT TTG CAT TAC TTA CGA CA-3′) and Agd4 (5’-CTG TAG TGA TAG GAA ATT TA-3′) amplify the resistant sequence of 195 bp and the sensitive sequence of 137 bp, respectively. These three bands of different sizes are easily resolved on 1.5% agarose gel and thus enable the easy determination of the genotype of each mosquito.

#### *Ace-1*^*R*^ G119S mutation detection

The phenotypes for insensitive *AChE* G119S mutation was determined according to Weill et al. [27]. The DNA PCR amplified with the primers Ex3Agdir (5′-GAT CGT GGA CAC CGT GTT CG-3′) and Ex3Agrev (5′-AGG ATG GCC CG CTG GAA CAG-3′) for an initial denaturation step of 3 min at 94 °C, followed by 35 cycles (94 °C for 30 s, 62 °C for 30 s and 72 °C for 20 s). After the final cycle, the products were extended for 5 min at 72 °C. The PCR fragments were then digested with *Alu*I restriction enzyme and fractionated on a 2% agarose gel. The two primers produced a 403 bp fragment, which is undigested by *Alu*I for susceptible homozygous mosquitoes (SS), and cut into two fragments (253 bp and 150 bp) for homozygous resistant (RR). Heterozygous individuals (RS) display a combined pattern.

### Data analysis

The WHO criteria were adopted for distinguishing between resistance/susceptibility status of the tested mosquito populations [19]. When less than 90%, mortality was observed, the population was considered ‘resistant’; between 90 and 97% of mortality the population was suspected ‘resistant’; and between 98% and 100%, the population was considered ‘susceptible’. GraphPad Prism 5.1 software was used to compare biochemical assay data [enzymatic activity per mg protein, levels of MFO, NSE and GST between Kisumu and each of the five sites *An. gambiae* (*s.l*.)] using the Mann-Whitney non-parametric U-test and to calculate the mortality rates with 95% confidence intervals. Allelic frequencies of L1014F *kdr* and *ace-1* G119S mutations were analyzed using Genepop version 4.0.10 [28]. To assess if the mutation frequencies were identical between survivors and dead in same population, the test of genotypic differentiation was performed [29]. Statistical significance was set at the 5% level.

## Results

### Insecticide susceptibility

The mortalities induced by all tested insecticides on *An. gambiae* (*s.l.*) from our study sites are indicated in Table 1. The mortality rates of the *An. gambiae* Kisumu susceptible strain was 100% for all tested insecticides, confirming the efficacy and quality of the impregnated papers. Based on the WHO criteria, the *An. gambiae* (*s.l.*) from our localities displayed resistance to six of the eight tested insecticides: pyrethroids (permethrin, deltamethrin and α-cypermethrin), organochlorine (DDT) and carbamates (bendiocarb and carbosulfan). Mortality rates ranged between 0 and 73%, which are far below the susceptibility threshold of 98%.

**Table 1:** Bioassay mortality of the field population of *An. gambiae* (*s.l.*) and Kisumu strain

Insecticide	Kisumu			Allokokro			Petessou			Dar Es Salam			Kennedy			N’gattakro		
	*n*	% M	St	*n*	% M	St	*n*	% M	St	*n*	% M	St	*n*	% M	St	*n*	% M	St
Permethrin 0.75%	102	100	S	100	18	R	98	27.6	R	101	42.6	R	103	2.9	R	101	39.6	R
Deltamethrin 0.05%	105	100	S	126	33.3	R	102	25.5	R	106	54.7	R	103	17.5	R	100	72.8	R
α-cypermethrin 0.05%	106	100	S	101	8.9	R	96	4.2	R	101	30.7	R	97	20.6	R	103	33.0	R
DDT 4%	103	100	S	75	0	R	100	0	R	102	2.0	R	102	1	R	99	2.0	R
Bendiocarb 0.1%	102	100	S	100	47	R	102	2	R	98	34.7	R	101	24.8	R	103	49.5	R
Carbosulfan 0.4%	104	100	S	102	24.5	R	101	21.8	R	101	13.9	R	99	14.1	R	102	15.6	R
Chlorpyri-methyl 0.4%	100	100	S	102	100	S	100	100	S	105	99	S	102	100	S	100	100	S
Pirimi-methyl 1%	100	100	S	101	100	S	100	100	S	101	100	S	105	100	S	100	85.7	R

*Abbreviations: n* number of tested mosquitoes, *% M* mortality proportion; *St* resistance status, *S* susceptible, *R* resistant

For pyrethroid insecticides, the mortality rates were less than 43% for permethrin, 73% for deltamethrin and 33.5% for α-cypermethrin. The highest resistance was observed for DDT with mortality rates ranging between 0 and 2%. For the carbamate insecticides, the mortality rate was higher for bendiocarb than for carbosulfan in our study sites, except for Petessou where carbosulfan induced more mortality (21.2%) than bendiocarb (2.0%). However, all *An. gambiae* (*s.l.*) populations from the five sites were susceptible to the organophosphates, except for the N’gattakro area where the *An. gambiae* (*s.l.*) population was resistant to pirimiphos methyl with a mortality rate of 85.7%.

### Effects of PBO synergist on *An. gambiae* (*s.l*.)

Due to the strong resistance observed to permethrin, α-cypermethrin, deltamethrin (pyrethroids), bendiocarb and carbosulfan (carbamates) in all our study sites, a synergist assay with PBO was carried out to assess the potential role played by enzymes in resistance observed.

**Fig. 2:**
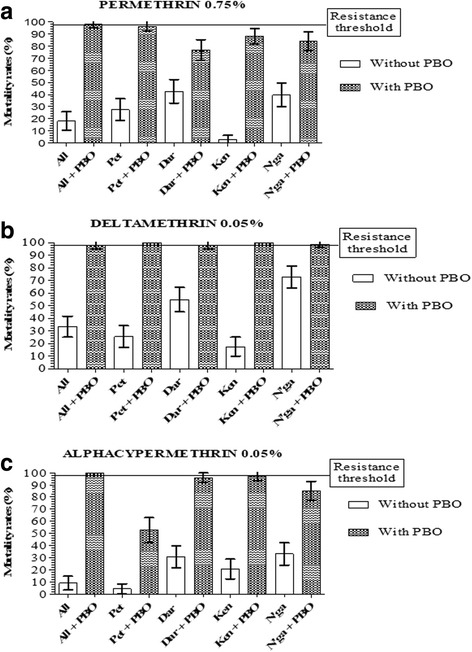
Mortality rate of *An. gambiae* (*s.l.*) from Bouaké to pyrethroids before and after 1 h of exposure to PBO: **a** permethrin; **b** deltamethrin; **c** alphacypermethrin. Error bars represent the 95% confidence interval. *Abbreviations*: All, Allokokro; Pet, Petessou; Dar, Dar Es Salam; Ken, Kennedy; N’ga, N’gattakro

**Fig. 3:**
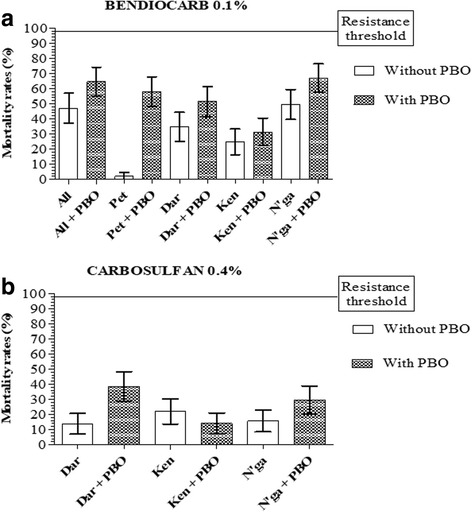
Mortality rate of *An. gambiae* (*s.l.*) from Bouaké to carbamates before and after 1 h of exposure to PBO: **a** bendiocarb; **b** carbosulfan. Error bars represent the 95% confidence interval. *Abbreviations*: Dar, Dar Es Salam; Ken, Kennedy; N’ga, N’gattakro

After pre-exposure to the PBO, the susceptibility to pyrethroids was restored in Allokokro. Mortality rates significantly increased from 18 to 98%, 33.3 to 98% and 8.9 to 100% for permethrin (Fisher’s exact test: *P <* 0.0001, OR = 223.2, CI = 50.29–990.9), deltamethrin (Fisher’s exact test: *P <* 0.0001, OR = 98, CI = 23.02–417.2) and alphacypermethrin (Fisher’s exact test: *P <* 0.0001, OR = 1918, CI = 110–33,450), respectively. In the others sites, susceptibility restoration was obtained with deltamethrin and a high increase of mortality rates with permethrin and alphacypermethrin (Fig. 2). The same trend was observed for carbamates (bendiocarb and carbosulfan) but to a lesser extent. We remind that only bendiocarb was tested with PBO in Allokokro and Petessou. Mortality rates significantly increased when pre-exposure to PBO from 47.0 to 64.5% in Allokokro (Fisher’s exact test: *P* = 0.0152, OR = 2.062, CI = 1.167–3.644) and from 1.7 to 58.0% in Petessou (Fisher’s exact test: *P <* 0.0001, OR = 69.05, CI = 16.11–295.9). A significant increase of mortality rates after PBO exposure was also observed with bendiocarb and carbosulfan in Bouaké district from Dar Es Salam and N’gatakro but not Kennedy. Mortality rates varied respectively for bendiocarb and carbosulfan, from 36.7 to 57.4% (Fisher’s exact test: *P* = 0.0225, OR = 1.995, CI = 1.131–3.521) and 13.9 to 38.3% (Fisher’s exact test: *P* = 0.0001, OR = 3.909, CI = 1.956–7.811) in Dar Es Salam, from 49.4 to 67.0% (Fisher’s exact test: *P* = 0.0154, OR = 2.070, CI = 1.173–3.655) and 15.6 to 29.6% (Fisher’s exact test: *P* = 0.0270, OR = 2.259, CI = 1.136–4.494) in N’gattakro, from 24.8 to 31.3% (Fisher’s exact test: *P* = 0.3492, OR = 1.390, CI = 0.7507–2.573) and 14.3 to 22.0% (Fisher’s exact test: *P* = 0.1972, OR = 0.5840, CI = 0.2793–1.221) in Kennedy (Fig. 3).

**Fig. 4:**
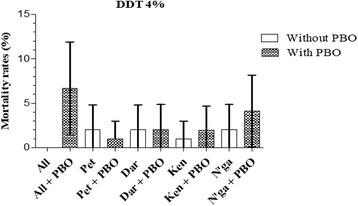
Mortality rate of *An. gambiae* (*s.l.*) from Bouaké to DDT before and after 1 h of exposure to PBO: Error bars represent the 95% confidence interval. *Abbreviations*: All, Allokokro; Pet, Petessou; Dar, Dar Es Salam; Ken, Kennedy; N’ga, N’gattakro

The pre-exposure to PBO did not change the mortality rates of *An. gambiae* (*s.l.*) to DDT in all sites (Fig. 4).

### Levels of detoxification enzymes associated with resistance

**Fig. 5:**
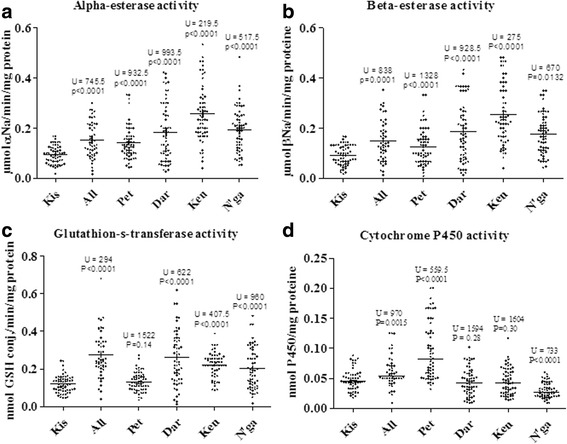
Activity profiles of non-specific α and β-esterases (**a**, **b**; OD = 550 nm), glutathione S-transferase (**c**; OD = 340 nm) and mixed-function oxidase (cytochrome P450) . (**d**; OD = 630 nm) in *An. gambiae* (*s.l.*) from Bouaké. *P* < 0.05 indicate significant increase in wild populations compared to values of Kisumu susceptible strain (Mann-Whitney tests). *Abbreviations*: Kis, Kisumu; All, Allokokro; Pet, Petessou; Dar, Dar Es Salam; Ken, Kennedy; N’ga, N’gattakro; OD, optical density

*An. gambiae* (*s.l.*) females of the five sites, unexposed to insecticides, were analyzed to evaluate their enzyme activities using biochemical assay. Figure 5 shows the distribution of enzymatic activity of non-specific esterases (α or β-naphthyl acetate), glutathione S-transferase and mixed function oxidase (cytochrome P450) compared to the susceptible reference Kisumu strain.

The mean esterase activities was significantly higher in *An. gambiae* (*s.l*.) from our five study sites (0.126–0.257 μmol α or β-naphthol/min/mg protein) than that in the reference Kisumu strain (0.092 μmol α or β-naphthol/min/mg protein) (all *P* < 0.05) (Fig. 5a and b).

The activities of GST of wild *An. gambiae* populations from our study sites (0.132–0.273 nmol GSH conj/min/mg protein) were significantly higher (all *P* < 0.05) than for Kisumu strain (0.119 nmol GSH conj/min/mg protein), except for Petessou (0.132; *P* > 0.05) (Fig. 5c).

For oxidases, the amount of P450 monooxygenases was only significantly higher for *An. gambiae* (*s.l*.) samples from Petessou and Allokokro (0.095–0.06 nmol P450/mg protein, respectively) than in Kisumu strain (0.047 nmol P450/mg protein) (*P* < 0.001). This level became significantly lower in *An. gambiae* (*s.l*.) populations from N’gattakro (0.029) compared to the Kisumu strain (*P* < 0.0001) and similar to the Kisumu strain in *An. gambiae* (*s.l*.) populations from Kennedy and Dar Es Salam (both *P* > 0.05) (Fig. 5d).

### Composition of *An. gambiae* species

**Fig. 6:**
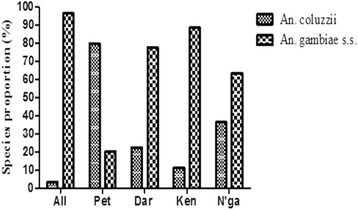
*An. coluzzii* and *An. gambiae* (*s.s.*) proportions in the different study sites. *Abbreviations*: All, Allokokro; Pet, Petessou; Dar, Dar Es Salam; Ken, Kennedy; N’ga, N’gattakro

Specific PCR assays showed the presence of *An. gambiae* (*s.s.*) and *An. coluzzii* in the five study sites. Except in Petessou where *An. coluzzii* was the dominant vector with a proportion of 79.7%, *An. gambiae* (*s.s.*) was the major species for the four other study sites representing 96.5% at Allokokro, 77.54% at Dar Es Salam, 88.64% at Kennedy and 63.64% at N’gattakro (Fig. 6).

### Resistance mechanisms

The L1014F *kdr* and *ace-1* G119S mutations were detected in both malaria vectors from Bouaké. High allelic frequencies L1014F *kdr* were detected in *An. gambiae* (*s.l.*) populations from five sites with frequencies between 0.83 and 0.99. The three genotypes for L1014F *kdr* (SS, RS and RR) were observed in alive as well as dead bodies of *An. coluzzii* and *An. gambiae* (*s.s.*). In the five sites, the L1014F *kdr* allele frequency was similar between both sub-groups of *An. coluzzii* (between 0.85 and 1 in alive group and between 0.79 and 1 in dead group,) and *An. gambiae* (*s.s.*) (between 0.97 and 1 in alive group and between 0.96 and 1 in dead group, Table 2).

**Table 2:** L1014F *kdr* mutation frequency of *An. gambiae* (*s.l.*) from Bouaké and association with phenotypic resistance from pyrethroid bioassays

	Phenotype	*An. coluzzii*					*An. gambiae* (*s.s.*)				
		*n*	RR	RS	SS	f (*kdrw*)	*n*	RR	RS	SS	f (*kdrw*)
Allokokro	Alive	4	–	–	–	–	70	70	0	0	1
	Dead	2	–	–	–	–	50	49	1	0	0.99
Petessou	Alive	51	37	13	1	0.85	1	–	–	–	–
	Dead	69	45	19	5	0.79	4	–	–	–	–
Dar Es Salam	Alive	19	17	2	0	0.95	55	53	2	0	0.98
	Dead	15	15	0	0	1	59	54	5	0	0.96
Kennedy	Alive	4	–	–	–	–	46	45	1	0	0.99
	Dead	5	–	–	–	–	27	27	0	0	1
N’gattakro	Alive	33	32	1	0	0.99	36	34	2	0	0.97
	Dead	19	19	0	0	1	53	51	2	0	0.98

*Abbreviations: n * number of females tested, *f* (*kdrw*), *L1014F kdr* mutation frequency within each species, *L1014F* a mutated allele from leucine to phenylalanine at codon 1014 of the para sodium ion channel gene, *SS* wildtype genotype, *RR* homozygous mutation, *RS* heterozygote for the *kdr* gene

*Note: kdr* mutation frequency do not differ significantly (*P* > 0.05) between dead and alive mosquitoes. Note that no L1014F *kdr* was detected. Frequencies were not calculated when the number of individuals tested was low (−)

In contrast, the low allelic frequencies *ace-1* G119S were observed in the *An. gambiae* (*s.l.*) population. Between the five study sites, the frequencies varied from 0.29 to 0.39. The *ace-1* G119S allelic frequency was significantly higher in alive in both species for samples of Allokokro [0.5 *vs* 0 for *An. coluzzii* and 0.49 *vs* 0.041 for *An. gambiae* (*s.s*.)] and in Dar Es Salam [0.67 *vs* 0.1 for *An. coluzzii* and 0.48 *vs* 0.26 for *An. gambiae* (*s.s*.)]. In Petessou, the same trend was observed in *An*. *coluzzii* (0.4 *vs* 0.16). In Kennedy *ace-1* G119S allele frequency of *An. gambiae* (*s.s*.) was higher for the alive (0.46 *vs* 0.17). In N’gattakro, there was no significant difference observed between the *ace-1* G119S allelic frequencies between the both sub-groups of *An. coluzzii* and of *An. gambiae* (*s.s*.) (Table 3).

**Table 3:** *ace-1*^*R*^ G119S mutation frequency of *An. gambiae* (*s.l.*) from Bouaké and association with phenotypic resistance from carbamate bioassays. Superscript ^a^ and ^b^ show a significant difference between alive and dead within the same population on a site (*P* < 0.05). Frequencies were not calculated when the number of individuals tested was low (−)

	Phenotype	*An. coluzzii*					*An. gambiae* (*s.s.*)				
		*n*	RR	RS	SS	f (*ace1*)	*n*	RR	RS	SS	f (*ace-1*)
Allokokro	Alive	–	–	–	–	–	49	2	44	3	0.49^a^
	Dead	–	–	–	–	–	49	0	4	45	0.041^b^
Petessou	Alive	49	0	39	10	0.4^a^	1	–	–	–	–
	Dead	40	0	13	27	0.16^b^	3	–	–	–	–
Dar Es Salam	Alive	9	3	6	0	0.67^a^	40	1	36	3	0.48^a^
	Dead	10	0	2	8	0.1^b^	29	0	15	14	0.26^b^
Kennedy	Alive	2	–	–	–	–	23	1	19	3	0.46^a^
	Dead	4	–	–	–	–	21	0	7	14	0.17^b^
N’gattakro	Alive	13	0	7	6	0.27	12	0	11	1	0.46
	Dead	4	0	4	0	0.5	21	0	9	12	0.21

*Abbreviations: f* (*ace-1*), *ace-1* G119S mutation frequency within each species, *G119S* mutated allele from glucine to serine at codon 119 of AChE gene, *SS* wildtype genotype, *RR* homozygous mutation, *RS* heterozygote for the *ace-1* gene

## Discussion

Previous studies on the insecticide resistance of malaria vectors around Bouaké conducted after the political crisis in Côte d’Ivoire were carried out with mosquitoes collected from the field sites used for experimental hut studies [12, 13]. These populations were found to be resistant to pyrethroids, carbamates and DDT. The present study showed the current level of resistance of malaria vectors in the natural environment (rural and urban) within Bouaké districts and two surrounding villages and the mechanisms involved.

Table 4 summarizes the levels of insecticide resistance and the detected mechanisms in *An. gambiae* (*s.l.*) populations. Resistance to pyrethroids, carbamates and organochlorines in *An. gambiae* (*s.l.*) populations was shown from the five study sites as usually observed throughout the Ivorian territory [13, 30–32] and in many African countries [33–35]. On the other hand, the populations of *An. gambiae* (*s.l.*) from the different study sites except those from N’gattakro, were susceptible to organophosphates. In the area of Bouaké, DDT and pyrethroids have been used in rice and cotton fields for long time. The wide use of these insecticides had lead to a selective pressure on untargeted flies, such as mosquitoes in aquatic stages [36–38]. The resistance observed in these sites seemed to be related to insecticide pressure from agricultural practices and vector control programs. Both activities are well known as the main sources of selection pressure on malaria vectors [39, 40]. The pre-exposure to PBO restored full or partial susceptibility to the three pyrethroids in the different sites. Khot et al. [20] showed that PBO was an oxidase and esterase inhibitor, which suggests the involvement of oxidases and to a lesser extent, esterases, in pyrethroids resistance observed in this study. These results were confirmed by biochemical tests which showed high levels of oxidase and esterase activity in Allokokro and Petessou and only high levels of esterase activity in Dar Es Salam, Kennedy and N’gattakro. A significant increase of glutathione-S-transferase activities was also detected in the different populations except for Petessou. This indicates that this enzyme was involved to a greater or lesser degree in the pyrethroid and DDT resistance observed. The role of metabolic resistance to pyrethroids has been already observed in malaria vectors from Côte d’Ivoire as well as neighboring countries [12, 41–43]. PCR results showed the presence of L1014F *kdr* and *ace-1* G119S mutations in the wild *Anopheles* population in Bouaké. The high L1014F *kdr* allelic frequency observed in these populations was also associated with these enzymes in the resistance to permethrin and alpha-cypermethrin observed in the four sites, except Allokokro. The strong resistance observed to DDT in Bouaké could be explained by the presence of the *kdr* mutation, which would probably be associated to glutathione S-transferase. [44, 45]. On the other hand, Petessou has the lowest glutathione-S-transferase activity (more or less equal to the levels of the susceptible strain), which suggests that this enzyme would not be associated to *kdr* mutation in DDT resistance in this locality. Pre-exposure to PBO resulted in a slight increase of mortality rate, reducing resistance in *An. gambiae* (*s.l.*) populations to carbamates. The mortality observed after PBO pre-exposure and the high level of oxidase and esterase activities suggest the involvement of a metabolic resistance in addition to the *ace-1* mutation. In this study, despite the absence of cross-resistance to carbamates and organophosphates, the presence of the *ace-1* mutation was confirmed, contrary the studies of Koffi et al. [12] in M’be where only metabolic resistance was detected. Edi et al. [46] showed that extreme and multiple resistance to carbamates in malaria control would be caused by coupling actions to CYP6 P450 enzymes and ace-1 duplication. Since ace-1 duplication was not studied in the present work further investigations would be necessary to better understand this mechanism.

**Table 4:** Insecticide resistance levels and resistance mechanisms involved in different *An. gambiae* (*s.l.*) populations

	Allokokro	Petessou	Dar Es Salam	Kennedy	N’gattakro
Perm	RR	RR	RR	RR	RR
Delta	RR	RR	RR	RR	RR
α-cyp	RR	RR	RR	RR	RR
DDT	RR	RR	RR	RR	RR
Bend	RS	RS	RS	RS	RS
Carbo	RS	RS	RS	RS	NT
Chlor	SS	SS	SS	SS	SS
Piri	SS	SS	SS	SS	RR
L1014F *kdr*	**++**	**++**	**++**	**++**	**++**
*ace-1R* G119S	**+**	**+**	**+**	**+**	**+**
NSE	**>**	**>**	**>**	**>**	**>**
MFO	**>**	**>**	**=**	**=**	**<**
GST	**>**	**=**	**>**	**>**	**>**

*Abbreviations: Perm* permethrin, *Delta* deltamethrin, *α-cyp* alphacypermethrin, *DDT* dichlorodiphenyltrichloroethane, *Bend* bendiocarb, *Carbo* carbosulfan, *Chlor* chlorpyri-methyl, *Piri* pirimi-methyl, *kdr* knockdown resistance, *ace-1* acetylcholinesterase-1, *NSE* non-specific esterases, *MFO* mixed-function oxidase (cytochrome P450), *GST* glutathione S-transferase, *SS* susceptible, *RS* intermediate, *RR* resistant, *NT* not tested, + low frequency, ++ high frequency (*kdr* and *ace-1*), **<** low activity, **=** similar activity, **>** high activity (compared to Kisumu)

The distribution of *An. coluzzii* and *An. gambiae* (*s.s.*)*,* agrees with previous findings that reported the presence of both species in Bouaké area [12, 13]. In urban areas, more than 63% of *An. gambiae* (*s.l.*) population consisted of *An. gambiae* (*s.s*.) while in rural area, *An. coluzzii* was predominant in Petessou at more than 79% and *An. gambiae* (*s.s.*) was predominant in Allokokro at more than 96%. In Bouake city, the main breeding sites of *Anopheles* species were rice fields. The predominance of *An. coluzzii* in Petessou could be due to the irrigated rice fields maintaining permanent breeding sites. It is known that *An. coluzzii* preferentially lay eggs in permanent or semi-permanent water collection sites usually associated with human activities, such as those created by irrigation, rice cultivation or polluted breeding sites in urban areas [47–49]. *Anopheles gambiae* (*s.s.*), on the other hand, preferentially uses rain-dependent and temporary breeding sites [50]. For the others four sites, vegetables are the main crops and rice cultivation is only practiced during rainy season. L1014F *kdr* and *ace-1 *G119S mutations had almost the same frequencies between both *An. coluzzii* and *An. gambiae* (*s.s*.). L1014F* kdr* allelic frequency was also similar between live and dead mosquitoes for both species, indicating that the resistance to pyrethroids was largely due to metabolic resistance, as shown in the results. In contrast, the *ace-1 *G119S mutation was observed in a very low proportion among dead mosquitoes of these vectors.

This study highlights the complex interaction of mechanisms conferring multiple resistances to chemical insecticides in malaria vectors from Bouaké. The results indicated that metabolic detoxification was the more efficient mechanism of pyrethroid resistance compared to target site insensitivity contrary to carbamates. In the context of alternative vector control tool development, it would be important to evaluate the involvement of each enzyme in the resistance of specific insecticide families or active ingredient.

## Conclusion

Two malaria vectors were identified in Bouake area, *An. coluzzii* and *An. gambiae *(*s.s*.). These vectors were strongly resistant to pyrethroids, carbamates and DDT. In this resistance, two mechanisms were involved: genes mutation (L1014F* kdr* and *ace-1* G119S) and metabolic resistance (detoxification enzymes). In this area, metabolic resistance would be the main mechanism involved in pyrethroid resistance especially for deltamethrin. This study demonstrated that PBO would impact on the efficacy of insecticides used for vector control. According to these results, it would be interesting to assess the effectiveness of long lasting nets, combining a pyrethroid with PBO in a large scale for the control of pyrethroid resistant malaria vectors in Côte d’Ivoire.

## Abbreviations

*ace-1*: Acetylcholinesterase-1
ACHE: Acetylcholinesterase
CTAB: Cetyl Trimethyl Ammonium Bromide
DDT: Dichlorodiphenyltrichloroethane
DNA: Deoxyribonucleic acid
GST: Gluthatione S-transferase
IPR: Institut Pierre Richet
IRS: Indoor residual spraying
*kdr*: Knockdown resistance
LLINs: Long-lasting insecticide nets
MFO: Mixed function of oxidase
NMCP: National Malaria Control Program
NSE: Non-specific Esterase
PBO: Piperonyl butoxide
PCR: Polymerase chain reaction
WHO: World Health Organization
WHOPES: World Health Organization Pesticide Evaluation Scheme
